# Cancer Patients’ and Survivors’ Perceptions of the Calm App: Cross-Sectional Descriptive Study

**DOI:** 10.2196/16926

**Published:** 2020-02-10

**Authors:** Jennifer Huberty, Megan Puzia, Ryan Eckert, Linda Larkey

**Affiliations:** 1 College of Health Solutions Arizona State University Phoenix, AZ United States; 2 Behavioral Research and Analytics, LLC Salt Lake City, UT United States; 3 Mays Cancer Center University of Texas Health MD Anderson San Antonio, TX United States; 4 College of Nursing and Health Innovation Arizona State University Phoenix, AZ United States

**Keywords:** cancer, cancer survivors, mindfulness, meditation, consumer behavior, mobile apps, health, mental health

## Abstract

**Background:**

There is a need for tools to decrease cancer patients’ and survivors’ long-term symptom burden. Complementary strategies, such as meditation, can accompany pharmacologic therapy to improve symptoms. Although support programs with targeted content have wider reach, higher adherence, and greater impact, there are no consumer-based meditation apps designed specifically for cancer.

**Objective:**

This study aimed to gather information to advise the development of a cancer-specific meditation app in a small convenience sample of cancer patients and survivors who currently use the Calm app.

**Methods:**

Adult cancer patients and survivors who are Calm users (N=82) were recruited through the Daily Calm Facebook page. Participants completed a Web-based survey related to Calm app use and satisfaction, interest in and ideas for a cancer-specific Calm app, and demographic characteristics. Open-ended responses were inductively coded.

**Results:**

Participants were aged between 18 and 72 years (mean 48.60 years, SD 15.20), mostly female (77/82, 94%), white (65/79, 82%), and non-Hispanic (70/75, 93%), and reported using Calm at least 5 times per week (49/82, 60%). Although rates of satisfaction with current Calm components were high (between 65/82, 79% and 51/81, 63%), only 49% (40/82) of participants used guided meditations that they felt specifically helped with their cancer-related symptoms and survivorship, and 40% (33/82) would prefer more cancer-related content, with guided meditations for cancer-specific anxieties (eg, fear of recurrence; n=15) and coping with strong emotions (n=12) being the most common suggestions. A majority of participants (51/82, 62%) reported that they would be interested in becoming a member of a Calm cancer community (eg, in-app discussion boards: 41/46, 89%; and social media communities: 35/42, 83%). Almost half of the participants (37/82, 45%) reported that they would benefit from features that tracked symptoms in concurrence with app usage, but respondents were divided on whether this information should be shared with health care providers through the app (49/82, 60% would share).

**Conclusions:**

Responses suggest ways in which the current Calm app could be adapted to better fit cancer patients’ and survivors’ needs and preferences, including adding cancer-specific content, increasing the amount of content focusing on coping with strong emotions, developing communities for Calm users who are cancer patients and survivors, and including features that track cancer-related symptoms. Given differences in opinions about which features were desirable or would be useful, there is a clear need for future cancer-specific apps to be customizable (eg, ability to turn different features on or off). Although future research should address these topics in larger, more diverse samples, these data will serve as a starting point for the development of cancer-specific meditation apps and provide a framework for evaluating their effects.

## Introduction

### Meditation and Cancer Symptoms

In 2018, approximately 1.7 million people were diagnosed with cancer in the United States [1]. Although therapeutic advances have increased cancer survivorship rates from 49% to 69%, the burden of long-term symptoms arising from cancer and cancer therapy among patients and survivors, such as fatigue, depression, pain, and sleep disturbance, is high [2,3]. There is an urgent need for tools that are engaging and easily accessible for cancer patients and survivors that can help to decrease this burden [3]. The growing population of cancer patients and survivors can benefit from complementary strategies to accompany pharmacologic therapy and better manage symptoms [4].

According to a systematic review of surveys conducted in 18 countries across Europe, North America, and Oceania, meditation is the most common complementary strategy for symptom management among cancer survivors [5]. Meditation can be classified as a mindfulness-based strategy, comprising purposeful focus on the present moment without judgment [6]. Findings indicate that mindfulness-based interventions can improve mood, attitude toward one’s ability to cope with pain, fatigue, sleep disturbance, and anxiety related to cancer symptoms [7]. These interventions are typically delivered through in-person visits with trained providers, which can be time consuming and costly [8-12]. Even programs that allow at-home participation often involve regular meetings that can be difficult to manage alongside fatigue and pain [13]. Simplifying access for cancer patients and survivors may decrease symptom burden.

### Smartphone-Based Meditation

Smartphone-based meditation apps are a resourceful and novel way of delivering meditation practices for symptom management to cancer patients and survivors [14]. Smartphone use is virtually universal among cancer patients and survivors [15,16], and this population reports willingness to use app-based guided meditation [17]. A systematic review published in 2016 found that there were 539 mobile apps related to oncology, 117 of which were targeted toward patients, but few (<6%) were explicitly supported by industry [18]. The use of mobile health (mHealth) tools has been suggested as a means of increasing the scalability of behavioral interventions, thereby allowing a wider reach than possible with in-person interventions [19]. One study recommended Web-based mindfulness-based guided meditation to provide an opportunity for participation in meditation that is otherwise unavailable to many underserved cancer patients and survivors [20].

Despite the growth and promise in using mobile apps to deliver interventions, most empirically supported apps have been developed by the investigator within the context of specific research [21]. Generally, these research-specific apps are not widely available or set up for planned commercial dissemination. A focus on commercially available apps with a broad client base, adapted for specific cancer patients’ and survivors’ needs, may allow for easier dissemination. However, there is a lack of evidence for using consumer-based mobile apps to improve symptoms in cancer patients.

A recent review found that there were approximately 150 mobile apps for cancer patients and survivors offering mostly educational content and information [22]. Only 5 apps that targeted cancer patients and survivors included some form of meditation content, and to our knowledge, none of them has been empirically evaluated to support their potential feasibility or effects on cancer patients and survivors [22-24]. A systematic review by Jongerius et al [25] identified only 2 meditation studies for cancer patients delivered via mobile apps, both focusing on breast cancer patients. One study used a consumer-based app, Headspace, but did not track app utilization [26]. The other study was not specifically a mobile app, instead adapting Mindfulness-Based Stress Reduction for iPad delivery, and was conducted only over 6 weeks without any follow-up [27].

There are more than 500 mindfulness-based mobile apps available to the consumer (eg, multiple types of meditation, breathing exercises, music and sounds, and movement) [28-30]. Calm is the most popular health and fitness app in the United States (more than 50 million downloads and 1.8 million subscribers), with more than 100 guided meditations to teach users the basics of meditation and how to incorporate meditation into one’s life, and also includes hundreds of programs for intermediate and advanced meditators [17,31]. The Seven Days of Calm (ie, introductory course) introduces the user to mindfulness meditation and meditation practices. Users also have access to a library of meditation content, with 3- to 35-min guided meditations addressing a wide range of topics, including coping with negative emotions, increasing compassion and gratitude, and living in the present moment. Users also receive a new 10-min guided meditation every day (ie, The Daily Calm). Beyond basic guided meditations, Calm also offers a variety of other features, including relaxing, soothing music and nature sounds (Calm music), narrated fictional stories for sleep (Sleep Stories), breathing training exercises (Breathe Bubble), in-depth audio series providing education about living mindfully (Calm Masterclass), and video lessons on slow, mindful movement routines (Calm Body).

Despite the variety of content offered and a basis in scientifically validated clinical stress reduction theories [32-34], Calm was designed for the general population, not specifically targeted for cancer patients and survivors. Research suggests that programs targeted to specific patient groups have wider reach, higher adherence rates, and greater impacts on health behaviors [35,36]. The Center for eHealth Research and Disease Management recommends that to improve the uptake of electronic health (eHealth) on patient populations (eg, cancer), the patient or user feedback should be incorporated to help facilitate the development of interventions to be targeted for end users [37]. Targeting content and features in an app such as Calm provides researchers and clinicians with an established platform to disseminate smartphone-based meditation practices to cancer patients and survivors. The large reach and sustainability (ie, low cost, easy to access, and convenience to use any time or place) of consumer-based apps, such as Calm, may prove a viable and effective solution for patient and survivor symptom management if adapted using patient feedback. There is a need to explore cancer patients’ and survivors’ perceptions of consumer-based meditation apps and use this information to inform future content and features, making it possible for these apps to help improve the lives of those afflicted by cancer.

### This Study

The purpose of this study was to conduct formative research in a small convenience sample of cancer patients and survivors who use Calm to gather information (ie, perceptions, satisfaction, and suggestions) to advise the development of content and features that would better meet the needs and preferences of this population. Data from this study may inform future design for consumer-based apps that want to target specific populations and provide a framework for evaluating their effects within those populations.

## Methods

### Ethics Approval

The Institutional Review Board at Arizona State University (STUDY00010456) approved the study. All participants provided electronic consent before participating in the survey. The dataset generated or analyzed during the study is available from the corresponding author upon request.

### Study Design and Recruitment

This was a cross-sectional descriptive study. Participants were recruited from August 1 to 12, 2019, using a post on the Calm Facebook Community page asking users who had a past or current cancer diagnosis to participate in a survey. If interested, potential participants were provided a link to complete an eligibility screening confirming that they currently or previously had a cancer diagnosis and their age. Eligibility criteria were (1) having had a cancer diagnosis and (2) aged 18 years or older. Those who were eligible were able to move on to the survey questions (see Textbox 1). Before beginning the survey, participants were informed that their participation was voluntary, that they could stop the survey at any time, and that their responses were anonymous and would only be reported in aggregate. They were given the option to consent to participate in the study by clicking to continue to the survey questions on the next page.

### Survey

The survey was developed by 3 doctoral-level researchers and a master’s-level trained public health data analyst. The survey was Web-based and delivered via Research Electronic Data Capture (REDCap) and took approximately 15 min to complete. All responses were anonymous. Participants completed 21 questions developed by the research team to learn about usage of and satisfaction with the current version of Calm, interest in and ideas for a cancer-specific version of Calm, clinical and cancer characteristics (ie, current treatment status, cancer stage, type of cancer, and type of treatments received), and demographic characteristics. Survey questions are listed in Textbox 1; the complete survey, including response options for each question, is provided in Multimedia Appendix 1.

### Statistical Analysis

Quantitative data were analyzed using IBM SPSS version 25.0. Responses to open-ended questions were combined and categorized by first reviewing all responses, and the categories were developed inductively based on recurrent content within responses, defined and named, and then applied to responses [38]. Responses were individually coded by a master’s-level data analyst. To capture the breadth of participants’ feedback, themes were specific and reflected verbatim from participants’ responses. Responses that included content fitting multiple categories were assigned to all relevant themes. For example, referring to the types of new meditation content that could be created specifically for cancer patients and survivors that responded “Concentrating on gratitude and the positive things in your life, and also meditations dealing with anxiety when scheduled for follow-up testing” would be coded as *positive focus, gratitude* and *acute worrying about treatment sessions, scans, or appointments*. Not all participants completed every question; as such, the sample size differs across questions.

**Survey questions.**

**Calm usage characteristics**

1. How often do you use Calm?
2. For each component of Calm, please rank your level of satisfaction.
3. Are there any meditations in Calm that have been or were specifically helpful for your cancer-related symptoms or cancer survivorship?
4. Are there any meditations that were specifically not helpful for your cancer-related symptoms or cancer survivorship?
5. How do you feel about Calm’s content overall?

**Interest in Calm specifically for cancer**

1. Would you be more likely to use Calm if it was specifically made for cancer patients and survivors (ie, Calm for Cancer)?
2. Do you believe there could be components of Calm, other than what is already available, that could be specific for cancer patients and cancer survivors?
3. How useful do you find (Mindfulness Reminders, Tracking, and Share your Stats)?
4. What tools could Calm provide that would be useful to you, specifically related to having cancer or being a cancer survivor?
5. Calm currently has a Calm Community Facebook (FB) group. Are you a member of this group?
6. Would you be interested in being a member of a Calm Cancer Community or connecting with other cancer patients and cancer survivors, specifically?
7. If you could share your progress with managing symptoms (eg, fatigue, pain, and stress) with your doctor through Calm, would you use this feature?
8. Is there anything else you would like to share with us?

**Clinical characteristics**

When were you first diagnosed with cancer?
What specific type of cancer are or were you diagnosed with?
What stage is your cancer in currently?
(i) Are you currently in treatment for cancer or have you received cancer treatment in the past?
  (ii) What treatment(s) are you currently receiving/did you receive?
  (iii) When did you start treatment or for how long did you receive treatment?

**Demographic characteristics**

1. What is your birthdate?
2. How would you describe yourself (with regard to race)?
3. Do you identify as Hispanic or Latinx?
4. What gender do you identify with the most?

## Results

### Demographic Characteristics

A total of 82 Calm users with a current or past diagnosis of cancer (ie, cancer patients and survivors) participated in the survey. Participants were aged between 18 and 72 years (mean age 48.60 years, SD 15.20). The majority of respondents identified as female (78/82, 94%), white (65/79, 82%), and non-Hispanic (70/75, 93%; see Table 1).

Multimedia Appendix 2 presents information about participants’ types of cancer diagnoses and treatments received. Breast cancer was the most common type of cancer (35/82, 43%). The average age at diagnosis was 44.91 years (SD 4.78; minimum 18 and maximum 69 years). Approximately half of the participants (40/78, 51%) reported that their cancer was in remission or that they were cancer free. At the time of the survey, approximately one-third of the sample was receiving treatment for cancer (29/82, 35%).

**Table 1:** Demographic characteristics of the sample.

Category		Value, n (%)	
**Race^a^** **(N=79)**			
	White		65 (82)
	Asian or Asian American		4 (5)
	Black, African American, or Native African		3 (4)
	Native Caribbean or Afro-Caribbean Islander		2 (3)
	Biracial or multiracial		2 (3)
	American Indian or Alaskan Native		1 (1)
	Other		3 (4)
**Ethnicity (N=75)**			
	Non-Hispanic or Non-Latinx		70 (93)
	Hispanic or Latinx		5 (7)
**Gender (N=82)**			
	Female		77 (94)
	Male		5 (6)

^a^For race, participants were given the option of selecting multiple responses, such that the total number of responses does not sum to 79.

### Usage of and Satisfaction With Calm’s Components

More than half of participants reported that they used Calm at least five times per week (49/82, 58%), and rates of satisfaction with Calm components were generally high (see Tables 2 and 3). The Daily Calm was the most popular component, with only 1 (1%) of 82 respondents reporting that they did not use it. When asked to rank their level of satisfaction, the Daily Calm also had the highest satisfaction rate, with 95% (78/82) of respondents reporting that they were *satisfied* or *very satisfied* with this component. Calm Body and Masterclass were the least used components, with 49% (38/78) and 44% (34/78) of respondents, respectively, reporting that they did not use them. Calm music had the lowest satisfaction rates, with 4 (5%) of 81 respondents reporting that they were either *dissatisfied* or *very dissatisfied*, and an additional 13 (16%) of respondents reporting that they were *neither satisfied nor dissatisfied*.

**Table 2:** Participants’ self-reported frequency of using Calm (N=82).

Frequency	Value, n (%)
Less than 1 time per week	5 (6)
1-2 times per week	13 (16)
3-4 times per week	15 (18)
5 or more times per week	49 (60)

**Table 3:** Participants’ satisfaction with current Calm components and content.

Component	Very dissatisfied, n (%)	Dissatisfied, n (%)	Neither satisfied nor dissatisfied, n (%)	Satisfied, n (%)	Very satisfied, n (%)	I do not use this component, n (%)
Daily Calm (N=82)	1 (1)	0 (0)	3 (4)	24 (29)	54 (66)	1 (1)
Meditations (N=82)	1 (1)	0 (0)	6 (7)	21 (26)	45 (55)	9 (11)
Calm Music (N=81)	1 (1)	3 (4)	13 (16)	22 (27)	29 (36)	13 (16)
Sleep Stories (N=82)	1 (1)	0 (0)	5 (6)	26 (32)	31 (38)	19 (23)
Breathe Bubble (N=79)	1 (1)	1 (1)	6 (8)	13 (16)	27 (34)	31 (39)
Calm Masterclass (N=78)	1 (1)	0 (0)	8 (10)	16 (21)	19 (24)	34 (44)
Calm Body (N=78)	1 (1)	0 (0)	9 (12)	16 (21)	14 (18)	38 (49)

Almost half (40/82, 49%) of the respondents reported that there were meditation options in Calm that were helpful for their cancer-related symptoms or survivorship. When asked to describe the meditation programs that were specifically helpful and explain how they helped, participants reported that guided meditation that focused on anxiety, gratitude, and stress were most helpful for cancer symptoms and survivorship (see Table 4). Participants reported that guided meditations about anxiety and stress helped them to decrease reactivity in acutely stressful or anxiety-provoking situations, such as before scans, doctor’s appointments, or treatment sessions. They expressed that these meditations helped them to notice their feelings (eg, worry and pain) during these stressful moments and redirect their attention to the present, focusing on their breath, which helped them feel more centered and grounded. Participants reported that guided meditations about gratitude also helped them move their attention away from their cancer and focus on positive things in their lives right now. Several participants noted that guided meditations about gratitude also helped them to appreciate the difficulties of others and brought about a sense of peace, reminding them that they are not alone.

**Table 4:** Participants’ reports of guided meditation content specifically helpful for cancer-related symptoms or survivorship (N=40).

Meditation content	Value, n
Anxiety	12
Gratitude	6
Stress	5
Letting go (eg, of fears and control)	5
Breathing meditation	3
Pain	3
Sleep meditation	2
Living in the moment	1
Grief	1

When asked to report whether participants enjoyed Calm’s current content overall and if they would prefer that there was more content related to cancer, 79% (65/82) of respondents reported that they enjoyed Calm’s current content. However, 40% (33/82) of participants reported that they would prefer additional cancer-related content, such as topics related to being a cancer patient (19/82, 23%), topics related to being a cancer survivor (25/82, 30%), or other topics related to cancer (22/82, 27%). Some of the participants who reported that they would prefer other cancer-related topics provided a description of the topics they would like to see (see Table 5). The most commonly described topics were strong emotions that arise during or after cancer (especially fear, shock, uncertainty, and isolation) and life after surviving cancer (eg, healing, trauma, and lifelong worry).

**Table 5:** Descriptions of other cancer-related guided meditation content that participants would prefer for inclusion in Calm (N=22).

Meditation content	Value, n
Strong emotions during or after cancer	6
Life after cancer	5
Acceptance and letting go	4
Positive focus and gratitude	4
Chronic illness	3
Acute worrying about treatment sessions, scans, or appointments	3
Visualizations about staying strong (eg, mountain in a storm)	2
Bereavement	1
Narrators who are cancer patients and survivors	1
Cancer and pets	1

### Interest in Cancer-Related Calm Content

More than one-third (31/82, 38%) of the participants reported that they would be more likely to use Calm if it were specifically made for cancer patients and survivors, and 73% (60/82) believed that there could be components of the app modified specifically for cancer patients and survivors, beyond what is currently offered. When asked about what kinds of meditation content could be developed specifically for cancer patients and survivors, what they would like to see, and how it would be different from what is currently available, guided meditations for cancer-specific worries or anxieties were the most common suggestions (see Table 6). In particular, 7 participants shared fear of cancer recurrence as an important topic. Other cancer-specific worries included waiting for scan results, going into treatment or surgery, anxiety about potential side effects, terminating treatment, and fear of death. After cancer-specific worries, the next most common suggestion was guided meditations for dealing with strong emotions that arise during cancer, especially fear and anxiety.

When asked what participants would like to see and how it would be different from what is currently available on Calm, the most common response was the inclusion of cancer-specific content while maintaining the structure of the current Calm app (see Table 7). The second most common response was to change the emphasis of the app to focus more on topics that are relevant to being a cancer patient or survivor (eg, strong emotions, focus on the present moment, and positivity) or to curate existing Calm content to create cancer-relevant compilations within the current app (eg, as a series or Masterclass).

**Table 6:** Participants’ suggestions for new guided meditation content for cancer patients and survivors (N=51).

Meditation content	Value, n
Fears and anxieties specifically related to cancer	15
Managing difficult and strong emotions	12
Short guided meditations to use before or during treatment or scans	7
Similar to current content but specifically addressed to cancer patients and survivors	7
Hope for the future	7
Loving and knowing your body	7
Noninternally focused (eg, focus on surroundings, not on the breath or body)	7
Coping with side effects of cancer and treatment	5
Healing	5
Living in the present	5
Life after cancer	4
Interactions with others and accepting their reactions	4
Grief and mourning	4
Positive self-image	3
Visualizations	3
Remaining positive	2
Breathing	2
Movement and getting outdoors	2
Guilt	2
Trust	2

**Table 7:** Participants’ suggestions for cancer-specific guided meditation content and app components (N=30).

Meditation content	Value, n
Including cancer-specific content	16
Emphasizing different topics (eg, strong negative emotions and focus on present)	9
Curating current Calm content into cancer-relevant series	7
Reformatting content (eg, shorter guided meditations and interactive components)	6
Including content that is more personal or personalized	5
Validation of differences in cancer experiences	5
Content and format that reflects the chronological *Cancer Journey*	3
Including content related to pain management	3
Including spiritual content	2

### Interest in Connecting With Other Cancer Patients and Survivors

When asked if they would be interested in becoming a member of a Calm cancer community or in connecting with other cancer patients and survivors who used Calm, almost two-thirds (51/82, 62%) of participants agreed. Respondents who showed interest were asked to select from a list of different forums or types of communities in which they would be interested in participating. Discussion boards (eg, blogs or chat rooms available through the app) and communities on social media (eg, Facebook and Instagram) were the most popular suggestions, with 89% (41/46) and 83% (35/42) of respondents reporting that they would be interested in these types of communities, respectively. The majority of these respondents showed interest in communities integrated into the Calm app, such as group meditation programs that allowed multiple users to meditate at the same time (33/47, 70%) and the ability to contact other users individually via app-based direct messaging (24/38, 63%).

When asked if they would be interested in other types of communities, 6 (27%) of 22 respondents responded positively. Of the 4 respondents who provided additional open-ended descriptions of other types of potential communities that they might be interested in joining, all expressed a desire for communities with a narrower target audience. Specifically, they proposed that there might be different communities for different types of cancer, such as communities focused on chronic pain; communities for friends, family members, and caregivers; and communities specifically focused on staying positive.

### Interest in Cancer-Related Tools or Features to Support App Engagement

Participants were asked about the usefulness of Calm’s in-app tools designed to support user engagement (see Table 8). Responses indicate that more than 80% of participants used the tracking features and mindfulness reminders (15/82, 18%, and 16/81, 20%, reported that they did not use and pay attention to the tracking features and the mindfulness reminders, respectively), and more than half of the respondents reported that these features were either *mostly* or *very useful.* Most participants reported that they did not use or pay attention to the Share your Stats feature (48/82, 59%), which allows users to post meditation progress on social media, and only 20% (16/82) found this feature to be useful.

**Table 8:** Participants’ reports of usefulness of in-app tools to support app engagement (N=82).

Tool	Not at all useful, n (%)	Mostly not useful, n (%)	Sometimes useful and sometimes not, n (%)	Mostly useful, n (%)	Very useful, n (%)	I do not use or pay attention to this tool, n (%)
Tracking	2 (2)	2 (2)	10 (12)	19 (23)	35 (43)	15 (18)
Mindfulness Reminders	1 (1)	1 (1)	11 (13)	23 (28)	31 (38)	16 (20)
Share your Stats	9 (11)	5 (6)	5 (6)	9 (11)	7 (9)	48 (59)

Participants were asked to select from a list of possible new features to indicate which tools Calm could provide that would be useful to them, specifically related to having cancer or being a cancer survivor (see Table 9). The most popular response was creating a cancer community within the Calm app (59/82, 72%), followed by the sending of text messages via Calm with charts or graphs that concurrently present data on their cancer-related symptoms and Calm usage that week (37/82, 45%). There was modest support for tools for tracking symptoms or Calm usage exclusively or sharing symptom or usage reports with health care providers.

Participants who selected *Other tools* were given the option to describe additional tools that were not listed but might be useful for cancer patients and survivors. A total of 3 respondents expressed a desire for notifications with positive affirmations, encouraging words, or inspirational quotes. Another participant noted that it could be beneficial if tools in which cancer patients and survivors shared information with their health care providers also allowed providers to respond with feedback. In addition, 3 respondents who were currently undergoing cancer treatment expressed concern about receiving notifications with information about their cancer symptoms, suggesting that this would bring additional, unnecessary attention to difficulties that they are already highly aware of.

**Table 9:** Participants’ reports about potential app-related tools specifically useful for cancer patients and survivors (N=82).

Tool	Reported helpful, n (%)
Calm Cancer Community (ie, engagement with others through the Calm app)	59 (72)
Weekly text messages with a report (ie, graph or chart) about your use of the app and how you are feeling (ie, you track your cancer specific symptoms in Calm)	37 (45)
Daily text messages with feedback related to how you are feeling (ie, you track your cancer-specific symptoms in Calm) with a weekly report (ie, graph or chart)	27 (33)
Share your weekly symptom report with your health care provider	21 (26)
Daily text messages with feedback related to the use of the app (ie, time spent using, etc)	17 (21)
Share your stats (ie, time spent using) with your health care provider	14 (17)
Other tools	6 (7)

### Interest in Symptom-Tracking Features

When participants were specifically asked if they would use a feature that allowed them to share progress with managing symptoms (eg, fatigue, pain, and stress) with their doctor using the Calm app, 60% (49/82) reported that they would. Participants who reported that they would use this feature shared their ideas about how the feature would function (see Table 10). Respondents shared that the feature would allow them to complete surveys about their cancer-related symptoms (eg, before or after a meditation session, n=7) and to use an in-app dashboard to create customizable reports about changes in their symptoms and app usage (n=4), which they could generate and then choose to share with their health care provider (n=12). Other individuals suggested that doctors could have more direct access to their symptom information, within the app (n=2), integrated into existing eHealth platforms within their health care systems (n=3), or through regular (eg, weekly and monthly), automatic emails that sent reports to providers (n=4).

**Table 10:** Participants’ ideas for symptom-tracking and symptom-sharing features (N=33).

Mechanism or ability	Value, n
User-generated reports to share with health care providers	12
Surveys about symptoms within the app	7
Regular reports automatically sent or emailed to health care providers	4
Dashboard to create customizable symptom and app usage tracking reports	4
In-app feature that allows personal contact with users’ health care provider	4
Integration with existing electronic health platforms within users’ health care networks	3
Regular reports automatically sent or emailed to users	2
Feature allowing health care provider to directly access users’ reports or data	2

Responses from participants who did not desire a feature allowing them to share their symptoms with their health care provider (33/82, 40%) indicated the primary reason was that it was easier or preferable to discuss symptoms or progress in person during visits (see Table 11). Others felt that this feature was unnecessary, as they already had a system in place for tracking their symptoms or had other means to easily contact their health care provider if they needed to discuss their symptoms.

**Table 11:** Reasons for not wanting to share symptoms with health care providers through Calm (N=23).

Reason	Value, n
Prefer to share symptoms with health care provider in person	10
Concerns about privacy or confidentiality	3
Already track symptoms with other methods or systems	3
Tracking and sharing symptoms is not relevant to current needs	3
Difficult or burdensome for self (eg, emotionally) or provider (eg, time)	3
Already satisfied with communication with health care provider	2
Not currently in treatment	1

## Discussion

### Principal Findings

The purpose of this study was to conduct formative research in a small convenience sample of cancer patients and survivors who use Calm to gather information (ie, perceptions and satisfaction) to advise the development of content and features that would better meet the needs of this population. This was the first study to assess perceptions of cancer patients and survivors who use a consumer-based mobile app for meditation. Data from this study may inform future design for consumer-based apps that target specific populations and provide a framework for evaluating their effects within those populations.

#### Interest in Cancer-Related Calm Content

Participants were highly satisfied with Calm and used the Daily Calm most frequently with high satisfaction. Calm’s guided meditations related to anxiety, gratitude, and stress were considered to be the most helpful for cancer symptoms and survivorship. However, half of the participants did not think that the guided meditations were specifically helpful for their cancer-related symptoms or survivorship, and 73% (60/82) felt that there could be components of Calm modified to be more specific to the experiences of cancer patients and survivors. Suggestions for new content were mostly related to managing difficult emotions and fears and anxieties related to cancer, enduring fears of recurrence, and loving their bodies during and after cancer. Participants were interested in being connected with other Calm users who were cancer patients and survivors. Importantly, having a support community within the Calm app was overwhelmingly the most commonly suggested in-app tool for supporting app engagement. Most participants wanted to share their Calm use and the management of their symptoms with their care providers, but some preferred to do so in person.

Calm content was well received by cancer patients and survivors. Specifically, participants appreciated the stress-, anxiety-, and gratitude-related contents. This is likely because cancer-related stress often persists well beyond the disease’s diagnosis and treatment [39,40]. These stressors include fear of recurrence, limitations in physical function, and experiences and recovery from major treatments (eg, chemotherapy and radiation). Even transitioning out of treatment (eg, fewer medical visits) can cause stress because patients have more responsibility in monitoring and managing their symptoms. At present, Calm has 154 total pieces of content related to stress (98 pieces), anxiety (113 pieces), and a combination of stress and anxiety (57 pieces). Calm provides an easy way for cancer patients and survivors to access content that can help them cope with stress and anxiety. Other cancer-specific meditation mobile apps should consider content related to stress and anxiety.

Gratitude or the ability to notice and appreciate the present moment and the positive aspects of one’s life plays an important role in cultivating and maintaining well-being [41]. Gratitude has also been shown to build resilience and to help individuals cope with stress and anxiety [42]. For example, studies in breast cancer patients using Web-based gratitude interventions or gratitude journaling have reported significant decreases in death-related fear, fear of reoccurrence, improvements in daily psychological functioning, greater use of adaptive coping strategies, and greater feelings of being supported by the people around them compared with control groups [43,44]. Calm currently has 57 pieces of content related to gratitude and may also be an effective way to manage cancer patient- and survivor-related stressors [40,45].

Those who felt Calm could offer more cancer-related content recommended guided meditations for coping with strong emotions that arise during cancer (eg, fear, shock, uncertainty, and isolation). In addition, respondents desired content addressing the unique challenges of life after cancer (eg, healing, trauma, and lifelong worry). There are few resources that have been specifically designed to support cancer patients as they transition out of cancer treatment or that specifically address ongoing needs of posttreatment survivors (eg, fear of recurrence, anxiety, impaired body image, fitting into their previous social roles, and fatigue) [46-48]. A recent systematic review identified 10 studies that used mHealth apps (only 1 used meditation) targeting breast cancer survivorship, concluding that there is some promise for these mHealth apps for weight loss, reducing stress, and improving the overall quality of life. More research is needed to confirm the benefits of mobile apps for cancer patients entering survivorship and the unique challenges associated with this. The Calm app currently provides an easily accessible platform from which both cancer patients and survivors could access some content related to fears, yet these are not cancer specific. Mobile apps for cancer patients and survivors may want to consider content related to fears and emotions associated with the transition from patient to survivor and the potential long-term burden of cancer survivorship.

#### Interest in Connecting With Other Cancer Patients and Survivors

It is not surprising that cancer patients and survivors using the Calm app want to connect with other users. It is important to note that the participants in this study were recruited on Facebook, biasing their response to social support. However, it is also well known and documented that cancer patients and survivors turn to social support to gather resources and to assist with the coping process, as they navigate their way through cancer treatment and survivorship [49,50]. Participants in the study cited that they wanted to have access to other cancer patients and survivors through the app. Cancer patients and survivors often turn to digital and social media–based support groups [51]. Digital media provide an attractive format through which to access support groups because of the greater potential for anonymity and the ability for the cancer patients and survivors to meet social and emotional needs that may not be met by friends and family who do not have cancer [49,50]. Preliminary work demonstrates the potential for the use of the Calm app to improve a range of symptoms among cancer patients and survivors (eg, sleep disturbance, fatigue, anxiety, and depression) [17,31]. Currently, Calm offers support through a Facebook page for users and allows users to share their meditations stats on social media (eg, minutes of meditation and days of meditation). Calm is a unique platform through which the delivery of potentially effective complementary care (ie, mindfulness meditation) *and* access to a support group for cancer patients and survivors via social media could be achieved. Mobile apps for cancer patients and survivors may want to consider ways to include a mechanism for social support among users.

#### Interest in Symptom-Tracking Features

More than 80% (67/82) of participants indicated that they used the meditation tracking components within the current Calm app, and most reported that they found these features to be useful. Approximately half of the participants felt that the ability to track cancer symptoms in concurrence with Calm usage would be helpful to them. In breast cancer patients, multiple studies support the feasibility and acceptability of using mobile apps to monitor symptoms related to sleep [52], daily functional activity [53], and mental health [54]. In addition, studies of cancer care providers’ perceptions of mHealth apps and their potential clinical applications suggest that providers believe real-time outcome tracking is a promising utility, as it is often difficult for patients to accurately recount changing symptom trajectories over time [23,55]. Future development of cancer-specific mobile meditation apps should consider tracking not only minutes and days of meditation but also self-perceived symptoms over time (ie, stress, anxiety, and happiness) and potential illustrations of how these are related to time spent in meditation. This could be an effective strategy for helping cancer patients and survivors, in particular, adhere to participating in Calm long term.

Interestingly, there were divergent opinions about whether symptom information collected through Calm be shared with their health care providers, with approximately 60% (49/82) of participants indicating that they would share information with their health care providers and 40% (33/82) indicating that they would not. Apps that allow cancer patients and survivors a way to share information directly with health care providers are limited. A recent review of mobile apps for cancer found that 29 of the 151 available apps included features that allowed cancer patients and survivors to track symptoms (mainly fatigue, pain, mood, nausea, and sleep), 21 included the ability to generate graphical summaries for personal use or to share with health care providers (eg, through email or at doctor’s visits) [22]. Only 4 apps allowed users to log in and send messages to their health care team.

To our knowledge, there is little research on the effects of patients or survivors tracking symptoms and sharing this information with their providers (either in person or using an app). A small study of cancer patients (N=9) reported that using an mHealth app with the ability to access, monitor, and share their health-related information (eg, access care-related information and sharing information during visits) with providers during visits was empowering [56]. However, studies assessing the feasibility of tracking cancer symptoms via mobile apps have not included information about sharing this information with cancer care providers. Despite the potential benefits of tracking and sharing cancer-related symptoms through an app, health care providers have noted concerns about the privacy, communication, and storage of sensitive patient data [23,55]. Although mHealth technology may benefit users by encouraging them to assume an active role in managing their health and allowing them to engage in a more collaborative relationship with their providers [57], this adds complexity to determining and understanding the ownership of patient records, a role that has historically been held by the health care institution. Changes in these roles give rise to questions about which data should be shared and who should be responsible for safeguarding these data [58]. In order for meditation apps to incorporate features that connect cancer patients and survivors to their health care team, both parties should feel confident in the security and confidentiality of personal health information [59] and understand the extent to which the privacy of these data are and are not protected (eg, under Health Insurance Portability and Accountability Act [HIPAA] rules) [60].

### Limitations

The findings of this study should be interpreted in light of its limitations. First, survey participants were recruited via the Calm Facebook page. Owing to the fact that respondents were already Calm users, satisfaction with Calm was likely higher than would be observed in nonusers or users who were less engaged. However, it is notable that a substantial number of participants who were already satisfied with Calm agreed that the app could include components that better address the needs of cancer patients and survivors; this may highlight the broader appeal of a cancer-specific meditation app (eg, if individuals who do not currently use Calm but may consider using it and if it were specific to cancer). Future studies should also collect additional data about the proposed features or components of the current app that respondents were dissatisfied with and their reasons for dissatisfaction, as this information will inform future apps and potentially contribute to their long-term adherence. In addition, survey respondents were already engaged with the Calm community on social media such that the desire to connect with other Calm users may be higher than would be expected in the overall user population. The generalizability of these results may also be affected by the small sample size. To increase the potential benefits of a cancer-specific meditation app, questions about the desired features (eg, means of connecting with other users) should be further explored in larger, more diverse samples of cancer patients and survivors. Future research should also extend to others affected by cancer, such as caregivers and health care providers, who may have unique needs that could be addressed through a meditation app for cancer.

### Conclusions

This was the first study to survey cancer patients and survivors who use Calm and participate in Calm’s Facebook group to explore the desirability of a cancer-specific meditation app and to collect information about the types of content and features that would be most helpful for these users. Respondents felt that the components, content, and tools in the current Calm app could be better tailored to meet the needs of cancer patients and survivors. There was a desire for content that addressed cancer-specific anxieties (eg, scan anxiety and fear of recurrence) and content that focused on coping with strong emotions. Many patients and survivors indicated that they would benefit from features that tracked cancer-related symptoms in concurrence with app usage, but respondents were divided as to whether this information could be shared with health care providers through the app. This highlights the need for future apps for cancer patients and survivors to be customizable such that the users have the ability to turn different features on and off. Although future research should address these topics in larger, more diverse samples, these data may serve as a starting point for the development of meditation apps for specific patient groups and provide a framework for evaluating their effects within the target populations.

## Appendix

Multimedia Appendix 1 Calm for Cancer survey questions.

Multimedia Appendix 2 Clinical cancer characteristics in sample.

## Abbreviations

eHealth: electronic health
HIPAA: Health Insurance Portability and Accountability Act
mHealth: mobile health
REDCap: Research Electronic Data Capture

## References

[1] Siegel RL, Miller KD, Jemal A (2016). Cancer statistics, 2016. CA Cancer J Clin.

[2] Prochaska JJ, Coughlin SS, Lyons EJ (2017). Social media and mobile technology for cancer prevention and treatment. Am Soc Clin Oncol Educ Book.

[3] Harrington CB, Hansen JA, Moskowitz M, Todd BL, Feuerstein M (2010). It's not over when it's over: long-term symptoms in cancer survivors--a systematic review. Int J Psychiatry Med.

[4] West HJ (2018). Complementary and alternative medicine in cancer care. JAMA Oncol.

[5] Horneber M, Bueschel G, Dennert G, Less D, Ritter E, Zwahlen M (2012). How many cancer patients use complementary and alternative medicine: a systematic review and metaanalysis. Integr Cancer Ther.

[6] Kabat-Zinn J, Hanh TN (2009). Full Catastrophe Living: Using the Wisdom of Your Body and Mind to Face Stress, Pain, and Illness.

[7] Kwekkeboom KL, Cherwin CH, Lee JW, Wanta B (2010). Mind-body treatments for the pain-fatigue-sleep disturbance symptom cluster in persons with cancer. J Pain Symptom Manage.

[8] Russell L, Orellana L, Ugalde A, Milne D, Krishnasamy M, Chambers R, Livingston PM (2018). Exploring knowledge, attitudes, and practice associated with meditation among patients with melanoma. Integr Cancer Ther.

[9] Boxleitner G, Jolie S, Shaffer D, Pasacreta N, Bai M, McCorkle R (2017). Comparison of two types of meditation on patients' psychosocial responses during radiation therapy for head and neck cancer. J Altern Complement Med.

[10] Victorson D, Hankin V, Burns J, Weiland R, Maletich C, Sufrin N, Schuette S, Gutierrez B, Brendler C (2017). Feasibility, acceptability and preliminary psychological benefits of mindfulness meditation training in a sample of men diagnosed with prostate cancer on active surveillance: results from a randomized controlled pilot trial. Psychooncology.

[11] Carlson LE, Subnis UB, Piedalue KL, Vallerand J, Speca M, Lupichuk S, Tang P, Faris P, Wolever RQ (2019). The ONE-MIND Study: Rationale and protocol for assessing the effects of ONlinE MINDfulness-based cancer recovery for the prevention of fatigue and other common side effects during chemotherapy. Eur J Cancer Care (Engl).

[12] Carlson LE, Tamagawa R, Stephen J, Drysdale E, Zhong L, Speca M (2016). Randomized-controlled trial of mindfulness-based cancer recovery versus supportive expressive group therapy among distressed breast cancer survivors (MINDSET): long-term follow-up results. Psychooncology.

[13] van Waart H, van Harten WH, Buffart LM, Sonke GS, Stuiver MM, Aaronson NK (2016). Why do patients choose (not) to participate in an exercise trial during adjuvant chemotherapy for breast cancer?. Psychooncology.

[14] Plaza I, Demarzo MM, Herrera-Mercadal P, García-Campayo J (2013). Mindfulness-based mobile applications: literature review and analysis of current features. JMIR Mhealth Uhealth.

[15] Raghunathan NJ, Korenstein D, Li QS, Tonorezos ES, Mao JJ (2018). Determinants of mobile technology use and smartphone application interest in cancer patients. Cancer Med.

[16] Girault A, Ferrua M, Lalloué B, Sicotte C, Fourcade A, Yatim F, Hébert G, di Palma M, Minvielle E (2015). Internet-based technologies to improve cancer care coordination: current use and attitudes among cancer patients. Eur J Cancer.

[17] Huberty J, Eckert R, Larkey L, Kurka J, Rodríguez de Jesús SA, Yoo W, Mesa R (2019). Smartphone-based meditation for myeloproliferative neoplasm patients: feasibility study to inform future trials. JMIR Form Res.

[18] Brouard B, Bardo P, Bonnet C, Mounier N, Vignot M, Vignot S (2016). Mobile applications in oncology: is it possible for patients and healthcare professionals to easily identify relevant tools?. Ann Med.

[19] Kumar S, Nilsen WJ, Abernethy A, Atienza A, Patrick K, Pavel M, Riley WT, Shar A, Spring B, Spruijt-Metz D, Hedeker D, Honavar V, Kravitz R, Lefebvre RC, Mohr DC, Murphy SA, Quinn C, Shusterman V, Swendeman D (2013). Mobile health technology evaluation: the mHealth evidence workshop. Am J Prev Med.

[20] Zernicke KA, Campbell TS, Speca M, Ruff KM, Flowers S, Tamagawa R, Carlson LE (2016). The eCALM trial: eTherapy for cancer applying mindfulness. exploratory analyses of the associations between online mindfulness-based cancer recovery participation and changes in mood, stress symptoms, mindfulness, posttraumatic growth, and spirituality. Mindfulness.

[21] Huberty J, Vranceanu A, Carney C, Breus M, Gordon M, Puzia ME (2019). Characteristics and usage patterns among 12,151 paid subscribers of the Calm meditation app: cross-sectional survey. JMIR Mhealth Uhealth.

[22] Adam R, McMichael D, Powell D, Murchie P (2019). Publicly available apps for cancer survivors: a scoping review. BMJ Open.

[23] Berkowitz CM, Zullig LL, Koontz BF, Smith SK (2017). Prescribing an app? Oncology providers' views on mobile health apps for cancer care. JCO Clin Cancer Inform.

[24] Lippman H (2013). How apps are changing family medicine. J Fam Pract.

[25] Jongerius C, Russo S, Mazzocco K, Pravettoni G (2019). Research-tested mobile apps for breast cancer care: systematic review. JMIR Mhealth Uhealth.

[26] Rosen KD, Paniagua SM, Kazanis W, Jones S, Potter JS (2018). Quality of life among women diagnosed with breast cancer: a randomized waitlist controlled trial of commercially available mobile app-delivered mindfulness training. Psychooncology.

[27] Lengacher CA, Johnson-Mallard V, Post-White J, Moscoso MS, Jacobsen PB, Klein TW, Widen RH, Fitzgerald SG, Shelton MM, Barta M, Goodman M, Cox CE, Kip KE (2009). Randomized controlled trial of mindfulness-based stress reduction (MBSR) for survivors of breast cancer. Psychooncology.

[28] Wei M (2015). Psychology Today.

[29] Mani M, Kavanagh DJ, Hides L, Stoyanov SR (2015). Review and evaluation of mindfulness-based iPhone apps. JMIR Mhealth Uhealth.

[30] Duraimani SL (2019). A cross-sectional and longitudinal study of the effects of a mindfulness meditation mobile application platform on reducing stress and anxiety. Int J Yoga.

[31] Huberty J, Eckert R, Larkey L, Joeman L, Mesa R (2019). Experiences of using a consumer-based mobile meditation app to improve fatigue in myeloproliferative patients: qualitative study. JMIR Cancer.

[32] Carmody J, Baer RA (2008). Relationships between mindfulness practice and levels of mindfulness, medical and psychological symptoms and well-being in a mindfulness-based stress reduction program. J Behav Med.

[33] Gethin R (1998). The Foundations of Buddhism.

[34] Hofmann SG, Asnaani A, Vonk IJ, Sawyer AT, Fang A (2012). The efficacy of cognitive behavioral therapy: a review of meta-analyses. Cognit Ther Res.

[35] Hurtado-de-Mendoza A, Graves KD, Gómez-Trillos S, Song M, Anderson L, Campos C, Carrera P, Ostrove N, Peshkin BN, Schwartz MD, Ficca N, Cupertino A, Gonzalez N, Otero A, Huerta E, Sheppard VB (2019). Developing a culturally targeted video to enhance the use of genetic counseling in Latina women at increased risk for hereditary breast and ovarian cancer. J Community Genet.

[36] Hou S, Roberson K (2015). A systematic review on US-based community health navigator (CHN) interventions for cancer screening promotion--comparing community- versus clinic-based navigator models. J Cancer Educ.

[37] Børøsund E, Mirkovic J, Clark MM, Ehlers SL, Andrykowski MA, Bergland A, Westeng M, Solberg Nes L (2018). A stress management app intervention for cancer survivors: design, development, and usability testing. JMIR Form Res.

[38] Braun V, Clarke V (2006). Using thematic analysis in psychology. Qual Res Psychol.

[39] Bower JE, Meyerowitz BE, Desmond KA, Bernaards CA, Rowland JH, Ganz PA (2005). Perceptions of positive meaning and vulnerability following breast cancer: predictors and outcomes among long-term breast cancer survivors. Ann Behav Med.

[40] Messer D, Horan JJ, Larkey LK, Shanholtz CE (2019). Effects of internet training in mindfulness meditation on variables related to cancer recovery. Mindfulness.

[41] O'Leary K, Dockray S (2015). The effects of two novel gratitude and mindfulness interventions on well-being. J Altern Complement Med.

[42] Duprey EB, McKee LG, O'Neal CW, Algoe SB (2018). Stressful life events and internalizing symptoms in emerging adults: The roles of mindfulness and gratitude. Ment Health Prev.

[43] Otto AK, Szczesny EC, Soriano EC, Laurenceau J, Siegel SD (2016). Effects of a randomized gratitude intervention on death-related fear of recurrence in breast cancer survivors. Health Psychol.

[44] Sztachańska J, Krejtz I, Nezlek JB (2019). Using a gratitude intervention to improve the lives of women with breast cancer: a daily diary study. Front Psychol.

[45] Altschuler A, Rosenbaum E, Gordon P, Canales S, Avins AL (2012). Audio recordings of mindfulness-based stress reduction training to improve cancer patients' mood and quality of life--a pilot feasibility study. Support Care Cancer.

[46] Cheng KKF, Wong WH, Koh C (2016). Unmet needs mediate the relationship between symptoms and quality of life in breast cancer survivors. Support Care Cancer.

[47] Rowland JH, Bast RC, Croce CM, Hait WN, Hong WK, Kufe DW, Piccart–Gebart M, Pollock RE, Weichselbaum RR, Wang H, Holland JF (2016). Cancer survivorship: new challenge in cancer medicine. Holland-Frei Cancer Medicine.

[48] Wu H, Harden JK (2015). Symptom burden and quality of life in survivorship: a review of the literature. Cancer Nurs.

[49] Kim E, Han JY, Moon TJ, Shaw B, Shah DV, McTavish FM, Gustafson DH (2012). The process and effect of supportive message expression and reception in online breast cancer support groups. Psychooncology.

[50] Bender JL, Jimenez-Marroquin M, Jadad AR (2011). Seeking support on Facebook: a content analysis of breast cancer groups. J Med Internet Res.

[51] Zhang S, Bantum EO, Owen J, Bakken S, Elhadad N (2017). Online cancer communities as informatics intervention for social support: conceptualization, characterization, and impact. J Am Med Inform Assoc.

[52] Min YH, Lee JW, Shin Y, Jo M, Sohn G, Lee J, Lee G, Jung KH, Sung J, Ko BS, Yu J, Kim HJ, Son BH, Ahn SH (2014). Daily collection of self-reporting sleep disturbance data via a smartphone app in breast cancer patients receiving chemotherapy: a feasibility study. J Med Internet Res.

[53] Egbring M, Far E, Roos M, Dietrich M, Brauchbar M, Kullak-Ublick GA, Trojan A (2016). A mobile app to stabilize daily functional activity of breast cancer patients in collaboration with the physician: a randomized controlled clinical trial. J Med Internet Res.

[54] Kim J, Lim S, Min YH, Shin Y, Lee B, Sohn G, Jung KH, Lee J, Son BH, Ahn SH, Shin S, Lee JW (2016). Depression screening using daily mental-health ratings from a smartphone application for breast cancer patients. J Med Internet Res.

[55] Kessel KA, Vogel MM, Schmidt-Graf F, Combs SE (2016). Mobile apps in oncology: a survey on health care professionals' attitude toward telemedicine, mHealth, and oncological apps. J Med Internet Res.

[56] Klasnja P, Hartzler A, Powell C, Pratt W (2011). Supporting cancer patients' unanchored health information management with mobile technology. AMIA Annu Symp Proc.

[57] Qudah B, Luetsch K (2019). The influence of mobile health applications on patient - healthcare provider relationships: a systematic, narrative review. Patient Educ Couns.

[58] Tran C, Dicker AP, Jim HS (2017). The emerging role of mobile health in oncology. J Target Ther Cancer.

[59] Bhuyan SS, Kim H, Isehunwa OO, Kumar N, Bhatt J, Wyant DK, Kedia S, Chang CF, Dasgupta D (2017). Privacy and security issues in mobile health: current research and future directions. Health Policy Technol.

[60] Helm AM, Georgatos D (2014). Privacy and mHealth: how mobile health apps fit into a privacy framework not limited to HIPAA. Syracuse Law Rev.

