# Supplementary material for: Cancer Patients’ and Survivors’ Perceptions of the Calm App: Cross-Sectional Descriptive Study
Source: JMIR Cancer. 2020 Feb 10;6(1):e16926. doi: 10.2196/16926 (PMC7055788; doi:10.2196/16926)
Supplement: Multimedia Appendix 1 [file cancer_v6i1e16926_app1.pdf]

How often do you use Calm?

- ○ Less than 1 time per week
- ○ 1-2 times per week
- ○ 3-4 times per week
- ○ 5 or more times per week

For each component of Calm, please rank your level of satisfaction.

	Very satisfied	Satisfied	Neither satisfied nor dissatisfied	Dissatisfied	Very dissatisfied	I don't use this component
Daily Calm	○	○	○	○	○	○
Sleep Stories	○	○	○	○	○	○
Meditations	○	○	○	○	○	○
Calm Music	○	○	○	○	○	○
Breathe Bubble	○	○	○	○	○	○
Calm Body	○	○	○	○	○	○
Calm Masterclass	○	○	○	○	○	○

Are there any meditations in Calm that have been/were specifically helpful for your cancer-related symptoms/cancer survivorship?

- ○ Yes
- ○ No

  [If Yes] Which meditations were specifically helpful to you? [Open-ended]
  [If Yes] Please describe how they have helped you. [Open-ended]

Are there any meditations that were specifically not helpful for your cancer-related symptoms/cancer survivorship?

- ○ Yes
- ○ No

  [If Yes] Which meditations were specifically not helpful to you? [Open-ended]
  [If Yes] Please describe how they were not helpful to you. [Open-ended]

How do you feel about Calm's content overall?

- ○ I enjoy it as is for the most part
- ○ I would prefer topics related to being a cancer patient
- ○ I would prefer topics related to cancer survivorship
- ○ I would prefer other topics related to cancer
  - [If Other] Please describe below. [Open-ended]

Would you be more likely to use Calm if it was specifically made for cancer patients/survivors (i.e., Calm for Cancer)?

- ○ Yes
- ○ No

Do you believe there could be components of Calm, other than what is already available, that could be specific for cancer patients/cancer survivors?

- ○ Yes
- ○ No
  - [If Yes] What new meditation content could be created specifically for cancer patients/survivors? [Open-ended]
  - [If Yes] What would you like to see? How would they be different? [Open-ended]

Calm incorporates reminders, tracking, and a share your stats features to help users stay consistent with their practice:

- Reminders can be set on the app.
- Tracking keeps a record of your minutes of meditations and sessions completed and is displayed after you meditate.
- Share your Stats allows you to post to social media that you used the app.

How useful do you find these tools?

	Very useful	Mostly useful	Sometimes useful and sometimes not	Mostly not useful	Not useful at all	I don't use/ pay attention to this tool
Mindfulness reminders	○	○	○	○	○	○
Tracking	○	○	○	○	○	○
Share your Stats	○	○	○	○	○	○

What tools could Calm provide that would be useful to you, specifically related to having cancer or being a cancer survivor?

- ☐ Daily text messages with feedback related to the use of the app (i.e., time spent using, etc.)
- ☐ Daily text messages with feedback related to how you are feeling (i.e., you track your cancer specific
- ☐ symptoms in Calm) with a weekly report (i.e., graph or chart)
- ☐ Weekly text messages with a report (i.e., graph or chart) about your use of the app and how you are feeling
- ☐ (i.e., you track your cancer specific symptoms in Calm)
- ☐ Calm Cancer Community (i.e., engagement with others through the Calm app)
- ☐ Share your stats (i.e., time spent using) with your health care provider
- ☐ Share your weekly symptom report with your health care provider
- ☐ Other tools

    [If Other] Please describe below. [Open-ended]

Calm currently has a Calm Community Facebook (FB) group. Are you a member of this group?

- ○ Yes
- ○ No

Would you be interested in being a member of a Calm Cancer Community or connecting with other cancer patients/cancer survivors, specifically?

- ○ Yes
- ○ No

[If Yes] What types of communities might you be interested in?

	Yes	No
Discussion boards (i.e., blogs or chat rooms available through the app)	○	○
Group, app-based meditations (i.e., meditate at the same time as other users)	○	○
Direct messaging through the Calm app (i.e., messaging individual users)	○	○
Social media (e.g., Facebook, Instagram)	○	○
Other communities	○	○

[If Other] Please describe below. [Open-ended]

If you could share your progress with managing symptoms (e.g., fatigue, pain, stress) with your doctor through Calm, would you use this feature?

- o Yes
- o No
  - [If Yes] How would you imagine this feature working? [Open-ended]
  - [If No] Why would you not want to share your symptoms with your doctor? [Open-ended]

Is there anything else you would like to share with us? [Open-ended]

When were you first diagnosed with cancer? [Open-ended]

What specific type of cancer are/were you diagnosed with? [Open-ended]

What stage is your cancer in currently?

- o Stage I
- o Stage II
- o Stage III
- o Stage IV
- o Remission
- o Other
  - o [If Other] Please describe. [Open-ended]

Are you currently in treatment for cancer?

- o Yes
- o No
  - [If No] Have you received cancer treatment in the past?
    - o Yes
    - o No

[If Yes] What treatment(s) are you currently receiving/did you receive?

- ☐ Chemotherapy
- ☐ Pharmaceuticals
- ☐ Radiation
- ☐ Other
  - [If Other] Please describe. [Open-ended]

[If Yes] When did you start treatment/For how long did you receive treatment? [Open-ended]

When is your birthdate? [Date]

How would you describe yourself?

- ☐ American Indian or Alaskan Native
- ☐ Asian or Asian-American
- ☐ Native Hawaiian or Pacific Islander
- ☐ White, European-American, or Caucasian
- ☐ Black, African-American, or Native African
- ☐ Arab or Non-Arab North African/Middle-Eastern
- ☐ Native Caribbean or Afro-Caribbean Islander
- ☐ Bi-racial or Multi-racial
- ☐ Other
- ☐ I prefer not to respond

  [If Other] Please describe. [Open-ended]

Do you identify as Hispanic or Latinx?

- o Yes
- o No

What gender do you identify with the most?

- o Female
- o Male
- o Transgender
- o Nonbinary
- o Other
- o Prefer not to say

  [If Other] Please describe. [Open-ended]
