# Supplementary material for: Cancer Patients’ and Survivors’ Perceptions of the Calm App: Cross-Sectional Descriptive Study
Source: JMIR Cancer. 2020 Feb 10;6(1):e16926. doi: 10.2196/16926 (PMC7055788; doi:10.2196/16926)
Supplement: Multimedia Appendix 2 [file cancer_v6i1e16926_app2.docx]

*Appendix Table 1. Types of participants’ cancer diagnoses (N = 82)*

| Type | *n* (%) |
| --- | --- |
| Breast | 35 (42.68) |
| Hematological | 6 (7.32) |
| Thyroid | 6 (7.32) |
| Colorectal | 5 (6.10) |
| Cervical or uterine | 4 (4.88) |
| Head and neck | 4 (4.88) |
| Melanoma | 3 (3.66) |
| Neuroendocrine | 2 (2.44) |
| Anal | 1 (1.22) |
| Bladder | 1 (1.22) |
| Brain | 1 (1.22) |
| Esophageal | 1 (1.22) |
| Ovarian | 1 (1.22) |
| Pancreatic | 1 (1.22) |
| Prostate | 1 (1.22) |
| Soft tissue | 1 (1.22) |
| Vulvar | 1 (1.22) |
| Not specified | 8 (9.76) |

*Appendix Table 2. Types of cancer treatments participants have received*

| Treatment type | Currently in tx  (*N* = 29), *n* (%) | Not currently in tx  (*N* = 53), *n* (%) | Overall sample  (*N* = 82), *n* (%) |
| --- | --- | --- | --- |
| Chemotherapy | 8 (27.59) | 27 (50.94) | 35 (42.68) |
| Pharmaceuticals | 14 (48.28) | 19 (35.85) | 33 (40.24) |
| Radiation | 4 (13.79) | 25 (47.17) | 28 (34.15) |
| Surgery | 4 (13.79) | 7 (13.21) | 11 (13.41) |
| Hormonal | 3 (10.34) | 0 (0.00) | 3 (3.66) |
| Other | 5 (17.24) | 15 (28.30) | 20 (24.39) |
